# Nutraceutical Properties of Syringic Acid in Civilization Diseases—Review

**DOI:** 10.3390/nu16010010

**Published:** 2023-12-19

**Authors:** Iga Bartel, Izabela Mandryk, Jarosław O. Horbańczuk, Agnieszka Wierzbicka, Magdalena Koszarska

**Affiliations:** 1 Institute of Genetics and Animal Biotechnology, Polish Academy of Sciences, 05-552 Jastrzębiec, Poland; i.bartel@igbzpan.pl (I.B.); j.horbanczuk@igbzpan.pl (J.O.H.); a.wierzbicka@igbzpan.pl (A.W.); 2 Faculty of Medicine and Health Sciences, University of Applied Sciences in Nowy Sacz, 33-300 Nowy Sacz, Poland; imandryk@ans-ns.edu.pl

**Keywords:** syringic acid, civilization diseases, bioactive compounds, antioxidants, phenolic acid

## Abstract

Civilization diseases account for a worldwide health issue. They result from daily behavioral, environmental, and genetic factors. One of the most significant opportunities to prevent and alleviate the occurrence of these diseases is a diet rich in antioxidants like polyphenols. This review paper is concentrated on syringic acid (SA), one of the representative compounds of phenolic acids subgroups. There are many in vitro and in vivo studies on SA that assess its pivotal effects on oxidative stress and inflammation parameters. It is effective on metabolic risk factors as well, including hyperglycemia, high blood pressure, and hyperlipidemia. SA is one of the prominent polyphenolic compounds that may help address health issues related to civilization diseases.

## 1. Introduction

In past years, knowledge of nutrigenomics research has significantly advanced. Due to the development of this scientific field, it is more effortless to understand the nutritional influence on human health. On the other hand, this development provides a great chance to prevent and support the treatment of many diseases, especially diet-related diseases such as diabetes type 2, cardiovascular diseases (CVDs), and obesity [1]. These diseases, representing civilization diseases (also referred to as “lifestyle diseases” or “non-communicable diseases”), account for a global health problem. Many of them could be eliminated by maintaining proper daily behavior linked to a balanced diet. Providing bioactive compounds may exert a favorable effect on preventing the risk of civilization diseases. The prominent groups of bioactive compounds are phenolic acids, which belong to polyphenols that are widely distributed in plants. Phenolic acids are found in fruits, vegetables, whole grain products, and beverages such as green and black tea and coffee. One crucial example of phenolic acids is syringic acid (SA). This review article focuses on the role of SA as a pivotal factor in reducing the risk of developing civilization diseases by exerting a positive effect on metabolic parameters.

## 2. Civilization Diseases

Alarming data show that the number of people suffering from civilization diseases is continuously growing. Civilization diseases, also known as lifestyle diseases, are a group of chronic diseases. Their global increase has been escalating since the beginning of the 21st century. According to the World Health Organization (WHO), civilization diseases are the leading cause of premature death in 74% of the world’s population. First on the list are CVDs, such as stroke and ischemic heart attack, which cause 17.9 million deaths per year. The second place is occupied by cancers, causing 9.3 million deaths per year. Following closely are chronic respiratory diseases, which are responsible for 4.1 million deaths annually, and diabetes, which accounts for 2 million deaths per year [2]. The morbidity of civilization diseases in the majority of the population affects both developed and developing countries. It is linked to various factors such as genetic, socioeconomic, physiological, and environmental factors, and to daily behavior, including nutrition [3]. Also, urbanization and industrialization transformations have pivotal significance due to the changing appearance of diseases. Although the urbanization process has many advantages, it leads to serious problems, including overpopulation, air pollution, contaminations in the water, and deteriorated food quality [4]. These factors play an important role in the development of civilization diseases. Many relevant studies have shown that urbanization is highly correlated with the morbidity of cancers, diabetes, and obesity [5,6,7]. The progression of urbanization has led to meaningful changes in daily behavior. Notably, such changes have been observed when it comes to diet (which may be an imbalanced scheme of nutrition and poor in beneficial compounds), insufficient physical activity, and destructive habits, including smoking and drinking alcohol [8]. All of these factors promote negative changes in metabolic parameters, such as high blood glucose and high blood pressure. The lipid profile is also unfavorably changed, including excess total cholesterol and LDL-cholesterol, while HDL-cholesterol is too low.

## 3. Polyphenolic Compounds

**Figure 1:**
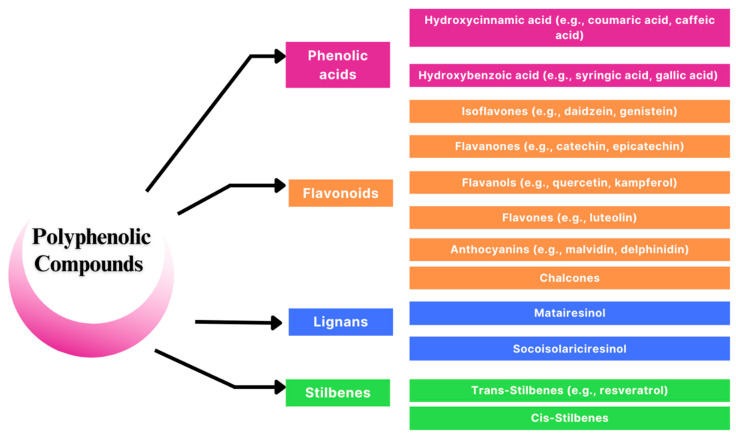
Subgroups of polyphenolic compounds [15,16,17].

Among the most prominent plant food compound groups are polyphenols. There is accumulating evidence that these bioactive substances are helpful for the proper functioning of the human body and are significant in the prevention of civilization diseases [9]. Polyphenols are secondary metabolites of plants that protect against negative external factors related to stressful environmental conditions. They are formed from primary metabolites. The latter include carbohydrates, amino acids, and lipids [10]. The presence of polyphenols ensures a defense reaction and response that is essential for survival. The group of harmful biotic and abiotic factors is wide and includes, for example, attacks of insects or pathogens (viruses, bacteria, etc.), herbivores, ultraviolet radiation, oxidants, saline stress, unfavorable temperature, and pH conditions [11]. Polyphenols are responsible for color, smell, and flavor in plant food. These compounds are found mainly in fruits, vegetables, legumes, and whole-grain food. They are also identified in beverages such as tea, coffee, cocoa, and red wine [12]. At present, the number of polyphenolic compounds derived from plants is calculated to be around 10,000, taking into account common features in chemical structures. Polyphenols are classified according to the number and combination of aromatic rings [13]. The aromatic feature and the highly conjugated system consist of numerous hydroxyl groups, allowing these compounds to act as effective donors of electrons or hydrogen atoms. As a result, they are able to neutralize free radicals and other reactive oxygen species (ROSs). Unlike aglycones, the majority of plant polyphenols are found in the form of glycosides, acylglycosides, and other conjugated forms. Under stressful conditions, enzyme hydrolysis can occur, converting glycosides to aglycones. This process is important because aglycones tend to be more active. The absorption of phenolic glycosides in human food products is lower than that of their corresponding aglycones in the digestive tract [14]. Therefore, the form of dietary polyphenols may influence the outcome of their health benefit efficacy. Polyphenols are divided into multiple subgroups (Figure 1).

**Figure 2:**
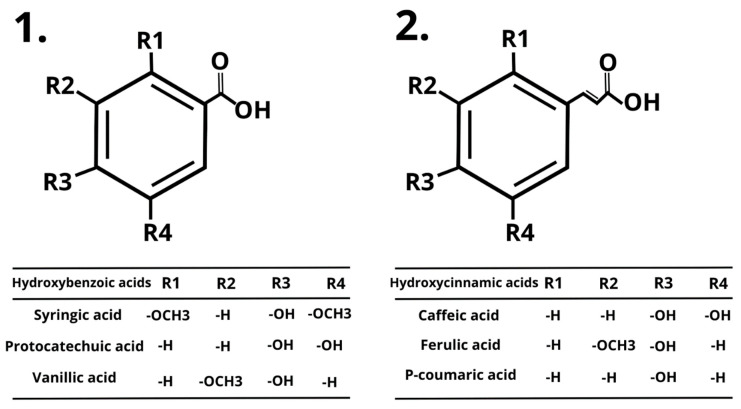
General structure of hydroxybenzoic acids, with examples, and hydroxycinnamic acids, with examples.

This wide group of compounds has not only demonstrated multiple functions for the plant growth but also a valuable impact on the improvement of human health status. Polyphenols are well-known as potent antioxidants, which are pivotal in many biological and pharmacological properties, including anti-cancer properties, anti-inflammatory properties, signaling molecules, anti-diabetic properties, and hepatoprotective properties [18,19,20,21,22,23]. The structure of phenolic acids consists of benzene rings, where hydrogen atoms are substituted by one carboxylic acid group and at least one hydroxyl group. In contrast to flavonoids, phenolic acids stand out, with better bioavailability and solubility in water [24]. They are divided into two subgroups—hydroxybenzoic acids and hydroxycinnamic acids (Figure 2), contributing to organoleptic characteristics like sour and bitter flavors [25]. Usually, they exist in bound forms, such as amides, esters, or glycosides, and they are seldom found in their free form [26]. They can also occur as combination with flavonoids, sterols, cell wall polymers, and many more examples, or be a part of different polyphenols such as anthocyanins and flavones [27].

Phenolic acids are identified in numerous products such as fruits, vegetables (especially in the skin and leaves), seeds (such as wheat seeds), strawberries (*Fragaria × ananassa Duchesne*), blueberries (*Vaccinium myrtilus*), bananas (*Musa* L.), chokeberries (*Aronia melanocarpa* (Michx.) Elliott), pomegranates (*Punica granatum*), blackberries (*Rubus* L.), and mangos (*Mangifera indica*). For example, among the hydroxycinnamic acids, the most abundant amount of caffeic acid (CA) is in fruits such as kiwi (*Actinidia*), but also in various products like coffee seeds, tobacco leaves, and olive oil. In turn, the cereals are a rich source of ferulic acid (FA). Generally, herbs, especially mint (Mentha ×piperita), oregano (*Origanum vulgare* L.), rosemary (*Rosmarinus officinalis*), basil (*Basileus*), thyme (*Thymus vulgaris*), and sage (*Lamiaceae*), contain phenolic acids, as do some beverages, including green and black tea and yerba mate. Furthermore, the content of hydroxybenzoic acids in plant food is not as high as it is in hydroxycinnamic acids, except for red fruits, black radishes (*Raphanus sativus var. sativus*), and onions (*Allium cepa* L.) [28,29,30,31].

## 4. Syringic Acid (SA)

SA belongs to the hydroxybenzoic acids subgroup. SA’s chemical structure comprises one benzene ring containing two methoxy (-OCH3) groups, one hydroxyl (-OH) group, and one carboxyl (-COOH) group. Furthermore, the presence of these methoxy groups at positions 3 and 5 on the benzene ring may contribute to its favorable biological properties. In turn, the hydroxyl group has influence on radical-scavenging activities [32]. Secondary metabolites, including SA, are formed via the shikimate pathway, which occurs in higher plants and microorganisms, but not in animals [33]. The main purpose of the shikimate pathway is to produce precursors essential for aromatic molecules, which are base substrates for both protein biosynthesis and the formation of polyphenolic compounds in plants [34]. In phenolic metabolism, many enzymes are involved, and the main metabolite is shikimic acid. This process is composed of seven stages, starting with an aldol-type condensation of phosphoenolpyruvic acid (PEP), derived from the glycolytic pathway, and D-erythrose-4-phosphate, sourced from the pentose phosphate cycle. The outcome is the formation of 3-deoxy-D-arabino-heptulosonic acid 7-phosphate (DAHP). Chorismic acid, the end product of this process, is a pivotal compound marking the culmination of the shikimate pathway. It serves as a crucial junction leading to post-chorismic acid pathways, facilitating the synthesis of L-phenylalanine, L-Tyrosine, and L-Tryptophan [35]. Phenylalanine plays a crucial role in the biosynthesis of SA, acting as a key compound for the conversion to hydroxycinnamic acids, including p-coumaric acid, CA, or FA. Further, the transformation into derivatives of hydroxybenzoic acids takes place via enzymatic β-oxidation reactions [36]. Many studies were conducted to demonstrate the various pharmacological impact of SA, but the safety and toxicity mechanism is still not confirmed in the literature [37]. A daily intake of 1–2 g of polyphenols is related to prevention of chronic illnesses [38], while phenolic acids should account for approximately 200 mg/d [24]. According to the available literature reports, the daily intake of phenolic acids is different in numerous countries, as follows: in Germany, the average is 222 mg [39]; in Finland, the average is 641 mg [40]; in France, the average is 599 mg [41]; and in Brazil, the average is 729.5 mg [42]. Similar differences are observed when it comes to the consumption of SA within Europe. The average expressed in mg/d is as follows: in the southern region, it is 3.427 ± 0.065; in the central region, it is 1.815 ± 0.062; and in the northern region, it is 2.118 ± 0.064 [43].

SA’s high content has been identified in many products such as olives (*Olea Europea*), pumpkins (*Cucurbita*), grapes (*Vitis vinifera* L.), blueberries (*Vaccinium myrtilus*), date palms (*Phoenix dactylifera* L.), walnuts (*Corylus avellana* L.), chard (*Beta vulgaris var. vulgaris*), acai palms (*Euterpe oleracea*), red wine, floral honey, and a number of other plants [36]. The particular SA contents are demonstrated in Table 1.

SA has multiple biomedical effects, including antioxidant, anti-inflammation, anticancer, anti-microbial, anti-diabetic, and hepatoprotective activities [58,59,60,61]. Moreover, it is a valuable compound in the industrial sector, due to being a part of the lignin, which is a plant cell wall component. SA seems to be a crucial substrate for the fungal laccase enzyme, demonstrating significant importance in bioremediation and in the pulp industry [62]. Due to its reducing properties, SA is useful as a dental-resin component in stomatology [63]. Natural derivatives of SA such as syringol, syringin, sinapine, canolol, and syringaldehyde are widespread in the plant kingdom. For example, syringaldehyde, a component of grapes and red wines stored in wooden barrels, is also found in wood smoke [64]. Baker et al., in their study, confirmed that acetosyringone, along with potentially other extracellular phenolics, may exhibit bioactive properties capable of impacting the interplay between plants and bacterial pathogenesis [65]. Another study, conducted by Zhou et al., revealed that SA contributed to the interaction between plants and soil microorganisms. SA hindered cucumber growth and modified rhizosphere microbial communities [66]. SA also demonstrated its photocatalytic ozonation activity under various operating conditions, with titanium dioxide as a photocatalyst [67]. SA is soluble in alcohols and ethers such as methanol, ethanol, and ethyl ether. In turn, solubility in water is relatively low. Although SA has strong antioxidant properties, in vivo studies have shown that its bioavailability is insufficient to achieve beneficial effects [68]. Hence, researchers are still seeking ways to increase its bioavailability, and among the promising carrier systems are liposomes, mainly nanoliposomes, which are modified using functional material [69]. Various techniques associated with nanoformulation have been researched, including cyclodextrin inclusion [70] and micelles [71].

## 5. Cardioprotective Effects of SA

Diabetic cardiomyopathy (DC) has emerged as a significant complication in individuals with diabetes. Sabahi et al. investigated the protective impact of SA on diabetes-induced cardiac injury in a rat model. Treatment with SA exhibited protective effects against diabetic cardiomyopathy by diminishing lipid peroxidation and protein carbonylation. These effects may be attributed to the antioxidant properties of this phenolic acid (Table 2) [72]. SA also demonstrated anti-hypertensive effects induced by N-nitro-L-arginine methyl ester (L-NAME) [73]. There is clear evidence that SA treatment may lower blood pressure and decrease lipid peroxides, while increasing NO availability and antioxidant levels in blood samples from rats [73]. Previous research evaluated the mechanism through which dietary quercetin (Q) might mitigate cardiac hypertrophy in the context of a fixed aortic constriction. A Q diet (including SA as a phenolic compound) reduced blood pressure and protected against damage in hypertensive rats [74]. SA was shown to reduce heart weight, fibrosis, and pathological cardiac remodeling in isoproterenol-treated mice. The same study demonstrated that SA downregulated Fn1 and collagen accumulation, but reduced the upregulation of Ereg, Myc and Ngfr. In isoproterenol-treated cells, SA lowers the upregulation of Fn1 and Nppb and also lowers cell size. This study confirmed the potential of SA as a beneficial agent in the treatment of cardiac hypertrophy and fibrosis (Table 2) [75]. Another study proved the cardio-protective effect of SA and resveratrol (RV) combined together, in rats, with isoproterenol (ISO)-induced cardio-toxicity. SA–RV pre-treatment significantly decreased serum CK-MB, LDH, and alkaline phosphatase, in contrast to cardiac tissue CK-MB, LDH, and SOD, CAT, the levels of which were increased by SA–RV pre-treatment. SA–RV together decreased levels of total cholesterol, triglycerides, low density lipoprotein cholesterol, very low-density lipoprotein cholesterol, and thiobarbutyric acid reactive substances and raised the level of density lipoprotein cholesterol in serum and in the heart. As well, the levels of NF-κB and TNF-α were significantly lowered by SA–RV [76].

All the above-described studies confirmed the cardioprotective properties of SA and showed the possibility of using it as a valuable agent in the fight against CVDs.

## 6. Anti-Cancer Properties of SA

Phytochemicals found in plants present innovative possibilities as potent drug agents in cancer therapy, owing to their lower toxicity and enhanced tolerance rates. Mihanfar et al. evaluated the effects of SA in vitro on human colorectal cancer cells (SW-480) and in vivo on colorectal cancer-induced rats. The in vitro study showed that SA treatment resulted in the inhibition of cellular proliferation, the induction of apoptosis through increasing cellular ROS and DNA damage levels, and the downregulation of major proliferative genes. In vivo observations, on the other hand, revealed a statistically significant decrease in both tumor volume and incidence when compared to the control group (Table 2) [61]. Velu et al. presented the mechanism of SA, extracted from *Alpinia calcarata Roscoe*, which mediated chemoprevention on 7,12-dimethylbenz(a)anthracene (DMBA)-induced hamster buccal pouch carcinogenesis (HBPC). The result of the study was the inhibition of the adverse changes in biochemical parameters of plasma and buccal mucosal tissues and also the downregulation of molecular markers expression (PCNA, Cyclin D1, and mutant p53) (Table 2) [79]. SA was also investigated in terms of cytotoxicity, oxidative stress, mitochondrial membrane potential, apoptosis, and inflammatory responses in gastric cancer cell culture (AGS). In that study, SA demonstrated anti-cancer activities by losing MMP, cell viability, and enhancing intracellular ROS. SA induced apoptosis in a selective, dose-dependent fashion by upregulating caspase-3, caspase-9, and poly ADP-ribose polymerase (PARP), while simultaneously downregulating the expression levels of p53 and BCL-2, lowering SOD, CAT, and GPx activities, suppressing gastric cancer cell proliferation and inflammation, and inducing apoptosis by upregulating mTOR via the AKT signaling pathway [98]. Lavanya et al. investigated the therapeutic benefits of SA on Wistar rats with induced hepatocellular carcinoma. Serum samples were employed to assess the levels of liver markers, while liver tissue samples were utilized for histopathological analysis and the evaluation of apoptotic and anti-apoptotic protein expression. It was shown that SA exhibited a protective effect against diethylnitrosamine (DEN)-induced hepatocellular carcinoma by reducing the serum liver marker levels and raising the expression of apoptotic proteins (Table 2) [80].

## 7. Anti-Diabetic Effects of SA

Type 2 diabetes (T2D) encompasses over 90% of all diabetes cases. T2D leads to many micro- and macrovascular complications, resulting in psychological distress for patients [99]. Many in vivo studies have demonstrated that SA has anti-diabetic properties in diabetic animals (Table 2) [81], and have also revealed that a group treated with SA exhibited better kidney histopathological outcomes compared to the diabetic group (Table 2) [82]. Moreover, Muthukumaran et al. induced T2D in Wistar rats by a single intraperitoneal injection of alloxan. The experimental group received SA orally for 30 days. Plasma glucose levels exhibited a notable decrease alongside a significant increase in plasma insulin and C-peptide levels; in addition, the aberrant levels of plasma and tissue glycoprotein components were restored to a state closely resembling normal (Table 2) [81]. Further studies on Wistar rats, in which T2D was also induced by the administration of alloxan, proved that SA restores the perturbed levels of carbohydrate metabolic enzymes, hepatic enzymes, and renal marker enzymes back to normal levels (Table 2) [83]. Another research group studied the effects of SA on renal, cardiac, hepatic, and neuronal diabetic complications in streptozotocin-induced neonatal (nSTZ) rats [84]. Treatment with SA decreased hyperglycemia, as well as the symptoms of polydipsia, polyphagia, and polyuria. Additionally, it reduced relative organ weight, cardiac hypertrophic indices, inflammatory markers, cell injury markers, glycated hemoglobin levels, histopathological scores, and oxidative stress. Furthermore, SA treatment increased Na/K ATPase activity (Table 2) [84]. Wei et al. showed that SA derived from the orchid *Herba dendrobii* is effective in inhibiting the progression of diabetic cataracts in both in vivo and in vitro rat models. SA has the capacity to downregulate the expression of AR and lens structural proteins at the mRNA level (Table 2) [85]. SA has been also shown, in in vitro conditions, to bind with the serum albumin and to prevent glycation-associated complications (Table 2) [86]. Using molecular modeling and mass spectrometric studies, the authors proved that Lys 93,261,232, Arg 194 and Lys 93, Arg 194 are the responsible binding residues for SA [86]. Wu et al. orally administered rats with lotus seedpod oligomeric procyanidins (LSOPCs) and further investigated the anti-glycative activity of LSOPC itself as well as that of its metabolites. SA was one of the metabolites detected in rat’s urine. These urinary metabolites exhibited antioxidant, anti-glycation, and carbonyl-scavenging properties (Table 2) [87]. All these results suggest that SA provides beneficial health effects in T2D treatment.

## 8. Anti-Inflammatory Effects of SA

The intricate processes of inflammation are orchestrated by an array of signaling molecules synthetized by immune cells such as leukocytes, macrophages, and mast cells [100]. It has been proven that SA exhibits anti-inflammatory, anti-obesity, and anti-steatotic properties [101]. Lee et al. studied isolated mouse peritoneal macrophages exposed to IFN-γ and LPS in vitro, with or without the presence of *Taraxacum coreanum* (TCC). Its chloroform fraction (SA and gallic acid (GA)) was employed for its anti-inflammatory properties [88]. In contrast to microphages without TCC treatment, macrophages treated with TCC in vitro exhibited significantly better inflammatory activation parameters, including the levels of iNOS, COX-2, IL-6, and TNF-α, and increased survival by 83% (Table 2) [88]. Another study presented by Costa et al. checked the anti-inflammatory properties of phenolic chemical compositions of *Eugenia *aurata** and *Eugenia punicifolia*. Those extracts include SA. As a result of ex vivo and in vivo trials, both extracts were observed to hinder neutrophil migration, suppress cell adhesion, and mitigate the degranulation processes [102,103]. Ham et al. used mice as an animal model to examine the effects of SA on obese diet-induced hepatic dysfunction. Obesity in mice was induced by supplementation with HFD over 16 weeks. In the experimental group fed with SA, the body weight was lower, and visceral fat mass, serum levels of leptin, TNF-α, IFN-γ, IL-6 and MCP-1, insulin resistance, hepatic lipid content, droplets, and early fibrosis were reduced. The circulation level of adiponectin was higher when compared to the control group. SA also downregulated lipogenic genes and inflammatory genes, but upregulated fatty acid oxidation genes in the liver (Table 2) [89]. Another study using rats with *carrageenan* induced *paw oedema* showed the anti-inflammatory properties of SA, as a component of *Hygrophila spinosa* leaf extract (Table 2) [90]. The in vitro, biophysical, and in silico studies conducted by Dileep et al. examined the inhibitory potential of specific benzoic acid derivatives, including SA, against secretory phospholipase A2 (sPLA2), a key enzyme within the inflammatory pathway. They unveiled a consistent binding mode within the active site of sPLA2 and exhibited inhibitory effects at micromolar concentrations [104]. These studies suggested that SA has a number of favorable anti-inflammatory applications.

## 9. Hepatoprotective Effects of SA

Hepatic disorders, acute and chronic, may result from various causes such as alcohol consumption (ALD: alcohol-induced liver disease; AFLD: alcoholic fatty liver disease) [105,106], obesity, metabolic syndrome (NAFLD: non-alcoholic fatty liver disease; NASH: non-alcoholic steatotic hepatitis) [107,108], high doses of drugs (DILI: drug-induced liver injury) [109,110], and autoimmune diseases (autoimmune hepatitis) [111]. In studies using animal models, SA has shown anti-inflammatory, anti-oxidative, and anti-pathogenic activities [36,59,75,91]. Using a mice animal model, Itoh induced chronic liver injury by injecting CCl(4) and concanavalin (ConA). This caused an increase in ALT and AST levels and also excessive deposition of collagen fibrils. Furthermore, the TNF-α, IFN-γ, and IL-6 in the bloodstream exhibited a swift increase [59,75]. At the next stage of the study, mice were administered SA intravenously. An analysis of liver sections demonstrated that SA effectively reduced collagen accumulation, markedly lowered the hepatic hydroxyproline content, and notably suppressed the cytokine levels (Table 2) [59,75]. Another study was performed by Ramachandran et al., in which the authors used rats as an animal model for hepatoxicity. Hepatoxicity was induced by the intraperitoneal administration of acetaminophen (APAP). Next, the rats were supplemented with SA by an oral route. The administered SA markedly reduced the levels of hepatic and renal function markers upregulated by APAP, bringing them closer to normal values (Table 2) [91]. In turn, the effect of SA on thioacetamide-induced hepatic encephalopathy was investigated by Okkey et al. In rats treated with SA inflammatory markers, levels were restored to normal. In addition, reduced oxidative stress and ammonia were observed. SA generally reduced inflammatory injury. The structures of astrocytes and hepatocytes were preserved in rats treated with SA. SA was also shown to restore behavioral impairments (Table 2) [76]. A recent study conducted by Somade et al. examined the effect of SA against methyl cellosolve (MECE)-induced hepatotoxicity in rats. Treatment with SA significantly reduced the levels of cytosolic Nrf2, known to be a nuclear transcription factor playing an important role in cellular defense against oxidative stress. It also stimulated the activities of the endogenous antioxidant enzymes (Table 2) [92].These findings, taken together, indicate that SA offers substantial protective effects against liver injuries.

## 10. Neuroprotective Effects of SA

SA has demonstrated a significant role in modulating excitatory neurotransmitters and alleviating behavioral dysfunctions within both the central and peripheral nervous systems. The treatment with SA holds promise for managing neurological dysfunction and behavioral impairments. Moreover, the proper administration and dosage of SA could be pivotal factors in the effective treatment of neurological diseases [112]. Rashedinia et al. examined the potential neuroprotective benefits of SA. Diabetic rats, upon receiving SA treatment, demonstrated remarkable enhancements in learning, memory, and motor deficits. Additionally, SA treatment significantly increased the mRNA brain expression of PGC-1α and NRF1, key regulators of energy metabolism, oxidative phosphorylation, and mitochondrial biogenesis. Furthermore, SA elevated the ratio of mtDNA to nDNA in both the brain and spinal cord of diabetic rats (Table 2) [93]. SA functioned as a protective agent by mitigating neuronal damage induced by cerebral ischemia in a rat model of aortic occlusion [94]. Biochemical investigations into the impact of SA administration in a rat model of brain ischemia revealed a reduction in oxidative stress and neuronal degeneration. SA caused the increase of SOD and NRF1, while increased MDA levels after ischemia were reduced after treatment in SA-treated rat brain (Table 2) [94]. Nonetheless, SA has been found to reduce the expression of BECN1 and caspase-3 in motor neuron cells of the spinal cord that are deceased or undergoing degeneration (Table 2) [95]. The impact of CA, SA, and a hybrid molecule combining both (CA–SA) on cerebral ischemic damage has been shown. CA–SA had a strong neuroprotective effect by the apparent inhibition of glia activation and cytokine and 5-LOX expressions in gerbil ischemic CA1 (Table 2) [96]. Rekha et al., in 2014, investigated the neuroprotective efficacy of SA on an MPTP/p-induced mouse model of Parkinson’s disease. Prior oral administration of SA was observed to enhance the impaired motor functions induced by MPTP/p by restoring catecholamine levels and antioxidant enzyme activity. Additionally, it ameliorated the expression of Th, DAT, and VMAT2, while significantly reducing the elevated expression of inflammatory markers induced by MPTP/p (Table 2) [97]. These mechanistic findings offer a partial explanation for the pharmacological effectiveness of SA as a potential therapeutic agent in the management of neurodegenerative disorders.

## 11. Conclusions

**Figure 3:**
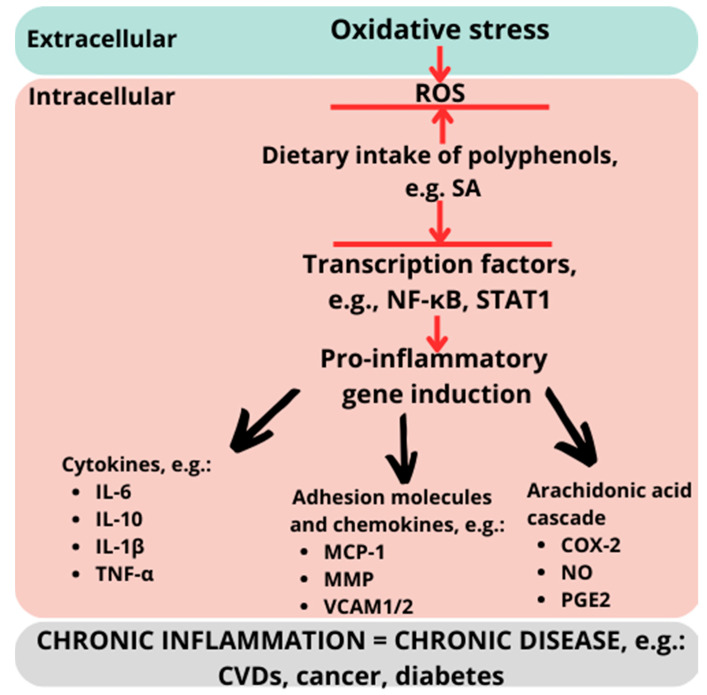
Anti-inflammatory effects of polyphenols.

It is common knowledge that our diets are currently based mainly on meat, dairy products, grain, and, above all, sugar. These types of food promote the rapid development of civilization diseases. This is one of the reasons that non-communicable diseases account for a real global problem. Unquestionably, it is necessary to seek opportunities to improve human health, especially when it comes to modifiable risk factors such as an imbalanced diet. A substantial dietary intake of polyphenols has been shown to reduce the risk of chronic diseases, particularly those associated with inflammation (Figure 3) [113,114]. Despite many studies, it is still unknown how, exactly, the anti-inflammatory mechanism of polyphenols works. Although in many animal studies polyphenols demonstrated strong anti-inflammatory effects, this did not always coincide with clinical trials [115,116,117,118,119]. While dietary polyphenols are not essential for humans, an increasing body of research has indicated that consuming products rich in polyphenols, e.g., fruits and vegetables, is advantageous for human health. This is particularly notable for individuals dealing with chronic inflammation, implying that polyphenols are vital bioactive dietary compounds for preventing and treating inflammation, as well as metabolic disorders [120,121,122]. Another issue concerns the dietary intake of polyphenols and their possible negative effects on humans and animals. Since there are no specific diseases caused by polyphenol deficiency, it is difficult to determine what the reference value of polyphenols intake should be for humans. The prevailing agreement suggests that regular consumption of polyphenol-rich products may be advantageous for individuals in mitigating or preventing inflammation and certain metabolic disorders. Despite many studies confirming the positive impact of polyphenols on the health of humans and animals, many published results show that high dosages of, e.g., resveratrol may have adverse effects such as hepatotoxicity [123].

SA is one of the prominent bioactive compounds identified in plant products that should be further investigated, due to its many positive effects. The covered studies greatly support the regular ingestion of SA for providing significant protection associated with a range of oxidative stress-related diseases such as hepatic and inflammatory diseases, T2D, neurodegenerative diseases, CVDs, and cancer. The biomedical effects of SA can be attributed to its robust antioxidant potential, owing to the presence of a phenolic structure with various functional groups. Phenolic acids, including SA, can be an effective aid in the fight against lifestyle diseases as a combined therapeutic strategy. Further research is needed to thoroughly investigate the molecular mechanism of natural compounds, both in the treatment and prevention of civilization diseases.

## Figures and Tables

**Table 1:** Content of SA in numerous products.

Product	Content	References
Alcoholic and non-alcoholic beverage [mg/100 mL]		
Dark beer	0.02 ± 0.02	[44]
Walnut liquor	3.12 ± 0.58	[45]
Red wine	0.27 ± 0.47	[46]
Green grape juice	0.05 ± 0.00	[47]
Traditional vinegar	0.70 ± 0.36	[48]
Food products [mg/100 g]		
Thyme	11.70 ± 0.42	[49]
Oregano	3.75 ± 5.30	[49]
Sage	3.35 ± 4.74	[49]
Rosemary	1.03 ± 1.79	[50]
Cloves	0.79 ± 0.00	[51]
Walnut	33.83 ± 13.96	[52]
Black olive	33.10 ± 32.13	[53]
Green olive	6.00 ± 8.49	[52]
Cauliflower	1.13 ± 0.02	[52]
Date (dried)	6.06 ± 3.81	[54]
Date (fresh)	2.45 ± 4.10	[54]
Currant	0.34 ± 0.13	[55]
Grape seed (*Cabernet Sauvignon*)	122.87 ± 0.25	[56]
Pumpkin pulp (*C. maxima ‘Bambino’*)	2.67 ± 0.05	[57]

**Table 2:** Potential uses of SA in civilization diseases.

Disease	Model	Dosage and Duration Period of Study	Effect of SA	References
CVDs	rats	100 mg/kg for 6 weeks	↓LDH, ↓CK-MB	[72]
		50 and 100 mg/kg for 6 weeks	↓cardiac TBARS, ↓carbonylated protein	[72]
	rats	50 mg/kg for 7 days	↓CK-MB, ↓LDH, ↓GGT, ↓hs-CRP, ↓SOD, ↓CAT, ↑NF-κB, ↑TNF-α	[77]
	mice	100 mg/kg/for 7 days	↓Ereg	[78]
	H9c2 cells	10 µL for 24 h	↓Nppa, ↓Nppb↓, ↓Col1a1 mRNA levels	[78]
cancers	rats	50 mg/kg for 15 weeks	↓tumor incidence, volume, and weight; ↓Gobblet cells	[79]
	SW-480 cells	1000–1200 µL for 48 h	↓CAT, ↓SOD, ↓GR, ↓PGx, ↓GST, ↓ERK1/2, ↓PI3K, ↓AKT, ↓NF-κB, ↓LC3, ↓BECLIN1, ↓ATG-3, ↑p53, ↑ROS, ↑apoptosis	[61]
	hamsters	50 and 100 mg/kg for 14 weeks	↓PCNA, ↓Cyclin D1, ↓mutant p53, ↓tumor incidence, Volume, and weight, ↑LPO, ↑CAT, ↑SOD, ↑GPx	[80]
	rats	25 mg/kg for 14 weeks	↓AFP, ↓AST, ↓ALT, ↓cellular expansion, nodules and hyperplasia, ↓BCL2, ↑Bax, ↑caspase 3, ↑cytochrome C	[80]
diabetes	rats	50 mg/kg for 30 days	↓plasma glucose, ↑plasma insulin, ↑C-peptide	[81]
	rats	50 mg/kg for weeks	↓blood glucose, ↓ALP, ↓TBARS, ↑GSH, ↑PGC-1α, ↑NRF1, ↑mtDNA/nDNA ratio	[82]
	rats	50 mg/kg for 30 days	↑insulin, ↑Hb, ↑glycogen, ↓glucose, ↓HbA_1c_	[83]
	rats	25 and 59 mg/kg for 10 weeks	↓hyperglycaemia, ↓polydipsia, ↓polyphagia, ↓polyuria, ↓relative organ weight, ↓cardiac hypertrophic indices, ↓inflammatory markers, ↓cell injury markers, ↓HbA_1c,_ ↓ROS, ↓histopathological score, ↑Na/K ATPase activity	[84]
	rats	200 µg/mL for 60 days	↓AR	[85]
	BSA glucose model system	50, 100 and 150 µg	↓structural alterations of BSA	[86]
	rats	300 mg/kg (LSOPC) for 1 day	↓AG	[87]
inflammation	mice	50, 100 and 200 mg/kg of TCC	↓TNF-α, ↓IL-6, ↓IL-1β	[88]
	mouse peritoneal macrophages	50, 100 and 200 µg/mL	↓iNOS, ↓COX-2, ↓NO, ↓PGE2, ↓IκBα, ↓MAPKs, ↓STAT1, ↓1KK phosphorylation	[88]
	Mice	HFD-SA diet 0.5g/kg for 16 weeks	↓body weight, ↓visceral fat mass, ↓serum levels of leptin, ↓TNF-α, ↓IFN-γ, ↓IL-6, ↓MCP-1, ↓insulin resistance, ↓hepatic lipid content, ↓droplets, ↓early fibrosis, ↓*Cidea*, ↓*Pparγ*, ↓*Srebp-1c*, ↓*Srebp-2*, ↓*Hmgcr*, ↓*Fasn,* ↓*Tlr4*, ↓*Myd88*, ↓NF-κB, ↑*Pparα*, ↑*Acsl*, ↑*Cpt1*, ↑*Cpt2* ↑circulation of adiponectin	[89]
	rats	200–400 mg/kg one dose of chloroform and alcoholic extracts of *Hygrophilia Spinosa*	↑anti-inflammatory activity	[90]
hepatic disorders	mice	10 mg/kg 2×/week for 4 weeks	↓MDA, ↓hepatic hydroxyproline content, ↓collagen accumulation, ↓ALT, ↓AST	[59]
	HSC	0.5 mg/mL	↓cells activation, ↓collagen genes, ↓α-SMA	[59]
	liver parenchymal hepatocytes	24 h long incubation in presence of SA	↑viability of hepatocytes	[59]
	mice	20 mg/kg, intraperitoneal administration	↓AST, ↓ALT	[75]
		10 mg/kg, intraperitoneal administration	↓TNF-α, ↓IL-6, ↓IFN-γ	[75]
	rats	25, 50, and 100 mg/kg for 6 days	↓AST, ↓ALT, ↓ALP, ↓GGT, ↓TBARS, ↓lipid peroxides, ↓SOD, ↓CAT, ↓GPx, ↑vitamin E, ↑vitamin C, ↑GSH	[91]
	rats	50 and 100 mg/kg for 14 days	↓AST, ↓ALT, ↓ALP, ↓LDH, ↓MDA, ↓ROS, ↑SOD, ↑GSH, ↓TNF-α, ↓IL-1β, ↓NF-κB, ↓IL-10, ↓iNOS, ↓8-OHdG, ↓GFAP, ↓ammonia concentration, preserved astrocyte and hepatocye structure	[76]
	rats	25, 50, and 75 mg/kg for 30 days	↓*Hmox1*, ↓*NQO1*, ↑SOD, ↑GST, ↑PGx, ↑NO, ↑CAT	[92]
		50 and 75 mg/kg for 30 days	↓MDA, ↓*Nrf2*, ↓*Keap1*	[92]
neurodegenerative diseases	rats	100 mg/kg for 6 weeks	↑learning, ↑memory, ↑motor coordination, ↑PGC-1α, ↑NRF1	[93]
		50 and 100 mg/kg for 6 weeks	↑mtDNA/nDNA, ↓lipid peroxidation, ↓inflammation, ↓demyelination in sciatic nerves,	[93]
	rats	10 mg/kg, intraperitoneal administration	↓MDA, ↓caspase-3-immunipositive neurons, ↓BECN1, ↓apoptotic neurons ↑SOD, ↑NRF1	[94]
	rats	10 mg/kg, intraperitoneal administration	↑SOD, ↑NRF1, ↓MDA, ↓caspase-3, ↓caspase-9	[95]
	Mongolian gerbils	20 mg/kg of CA-SA for 3 days	↓IL-1β, ↓5-LOX	[96]
	mice	20 mg/kg for 3.5 days	↑motor coordination, ↑neurochemicals, ↑TH, ↑DAT, ↑VMAT2, ↓IL-1β, ↓TNF-α, ↓COX-2	[97]

Abbreviations: ↑: increase, ↓: decrease, LDH: lactic acid dehydrogenase; CK-MB: creatine kinase MB; TBARS: thiobarbituric acid reaction substances; GGT: gamma-glutamyl transferase; hs-CRP: high sensitivity-C reactive protein; SOD: superoxide dismutase; CAT: catalase; NF-κB: nuclear factor kappa-light-chain-enhancer of activated B cells; TNF-α: tumor necrosis factor alpha; Ereg: epiregulin; Nppa: natriuretic peptide A; Nppb: natriuretic peptide B; Col1a1: collagen type I alpha 1 chain; GR: glutathione reductase; GPx: glutathione peroxidase; GST: glutathione S-transferase; ERK1/2: extracellular signal-regulated kinases ½; PI3K: phosphoinositide 3-kinases; AKT: protein kinase B; p53: cellular tumor antigen 53; LC3: microtubule-associated protein 1A/1B-light chain 3; ATG-3: autophagy related 3; ROS: reactive oxygen species; LPO: lipid peroxidation; PCNA: proliferating cell nuclear antigen; AFP: alpha-fetoprotein; AST: Aspartate transaminase; ALT: alanine transaminase; BCL 2: Bax: BCL-2 associated X protein; GSH: glutathione; ALP: alkaline phosphatase; PGC-1α: peroxisome proliferator-activated receptor-gamma coactivator; NRF1: nuclear respiratory factor 1; Hb: hemoglobin; HbA_1c_: glycated hemoglobin; AR: aldose reductase; BSA: binding sites in a model protein; NO: nitric oxide; PGE2: prostaglandin E2; COX-2: cyclooxygenase II enzyme; MAPKs: mitogen-activated protein kinases; STAT1: signal transducer and activator of transcription; LPS: lipopolysaccharide; TCC: Taraxacum coreanum chloroform fraction; IL-6: interleukin 6; IL-1β:interleukin 1β; IκBα: nuclear factor of kappa light polypeptide gene enhancer in B-cells inhibitor, alpha; IFN-γ: interferon gamma; MCP-1:monocyte chemoattractant protein-1; Cidea: essential transcriptional coactivator regulating mammary gland secretion of milk lipids; *Pparγ*: peroxisome proliferator-activated receptor gamma; *Srebp-1c*: Sterol regulatory element binding protein-1c; *Hmgcr*: 3-Hydroxy-3-Methylglutaryl-CoA Reductase; *Fasn*: fatty acid synthase; *Tlr4*: toll-like receptor 4; *Myd88*: myeloid differentiation primary response 88; *Acsl: acrocallosal syndrome*; *Cpt1 and 2*: carnitine palmitoyltransferase 1 and 2; HFD: high cholesterol diet; MDA: malondialdehyde; BECN1: beclin 1; 5-LOX: arachidonate 5-lipoxygenase; CA: caffeic acid; DAT: dopamine transporter; TH: tyrosine hydroxylase; HSC: hepatic stellate cells; iNOS: inducible nitric oxide synthase; 8-OHdG: 8-hydroxy-2-deoxyguanosin;, GFAP: glial fibrillary acidic protein; *Nrf2*: nuclear factor erythroid 2–related factor 2; *Hmox1*: heme oxygenase 1; *NQO1*: quinone oxidoreductase 1; *Keap1*: Kelch-like ECH-associated protein 1; VMAT2: vesicular monoamine transporter 2.

## Data Availability

Data sharing not applicable. No new data were created or analyzed in this study. Data sharing is not applicable to this article.
